# Saline Breast Implant Associated With Inflammatory Arthritis and Positive Antinuclear Antibodies (ANA): A Case Report

**DOI:** 10.7759/cureus.55061

**Published:** 2024-02-27

**Authors:** Emily R Littman, Kathleen Mccabe, Shazia Beg

**Affiliations:** 1 Medicine, University of Central Florida College of Medicine, Orlando, USA; 2 Rheumatology, University of Central Florida College of Medicine, Orlando, USA

**Keywords:** inflammatory arthritis, silicone, antinuclear antibodies, autoimmune disorder, breast implants

## Abstract

Breast implants, whether silicone or saline-filled, have a silicone shell and have been used for decades. Studies have shown an association between silicon with systemic lupus erythematosus, rheumatoid arthritis, progressive systemic sclerosis, and vasculitis. However, controversy and inconsistency have been pervasive in the literature with respect to the role of breast implants in the development of autoimmune diseases.

A 39-year-old female with a past medical history of breast cancer and a family history of Sjogren’s syndrome was referred to rheumatology for positive antinuclear antibodies (ANA) and polyarthralgia. She received textured saline breast implants for post-mastectomy reconstruction and subsequently developed fatigue, bilateral joint pain in her hands, wrists, and feet, and swelling in her fingers with prolonged morning stiffness, unintentional weight loss, and dry eyes. Physical examination revealed mild swelling of the bilateral metacarpophalangeal (MCP), proximal interphalangeal joint (PIP,) and distal interphalangeal (DIP) joints, and difficulty making a fist. Laboratory workup revealed a normal complete blood count (CBC) and comprehensive metabolic panel (CMP) with slight elevations in inflammatory markers. Autoimmune workup revealed positive ANA 1:640 (nucleolar) and 1:160 (speckled), positive U1RNP, and RNA polymerase III with negative SSA/SSB/dsDNA and Scl-70 Ab. Following elective implant removal after nationwide recall for heightened cancer risk, many of her symptoms spontaneously resolved.

The clinical case of inflammatory arthritis with positive ANA antibodies following saline breast implants highlights the importance of considering the possible health implications of silicone from a rheumatologic perspective. This case demonstrates that it may be reasonable that an association exists, and further research on a large scale would be valuable.

## Introduction

Breast implants, containing either silicone or saline, have been used for decades for reconstruction, augmentation, or revision purposes. Silicone implants containing a filling of silicone gel within a silicone shell were introduced in 1962 [1]. High rates of capsular contracture brought about the introduction of an alternative, saline-filled implant with a silicone shell three years later, however, silicone implants continued to be the predominant choice [2]. Over the years, due to various challenges with deflation, capsular contracture, and local failure, several incarnations of both types were developed [3]. In 1992, the United States Food and Drug Administration (FDA) became involved, as the number of health challenges in implant users increased and a 14-year moratorium on the use of silicone implants was imposed to allow for increased study [4]. Ultimately, no associations between silicone implant use and health challenges were found, the moratorium on silicone implants was lifted, and FDA approval was granted [5]. As of today, both silicone and saline implants have undergone many changes and continue to be widely used [2,3]. Both types have been the subject of much study, however, ongoing controversy about the implications of implant use on health persists [2,3].

No clear connections between implants and significant health issues, such as cancer, reproductive disorders, or connective tissue disease, have been established. Reports of rare T-cell lymphoma and other systemic illnesses have been reported so commonly enough in patients with breast implants, regardless of type, that the FDA issued a black box warning for the products in October 2021 [6,7]. Vague, systemic symptoms, such as headache, hair loss, fatigue, arthralgia, and cognition challenges, are common and the development of autoimmune conditions, including Sjogren’s syndrome and chronic fatigue syndrome, have been reported in implant recipients [8,9]. The myriad of complaints and their association with breast implants have been termed differently throughout the years, including ‘human adjuvant disease’ or ‘adjuvant breast disease’, ‘silicone-related symptoms complex’, and more recently, ‘autoimmune/inflammatory syndrome induced by adjuvants,’ also known as ASIA [10]. Research has yet to identify a concrete etiology for these symptoms, however, silicone implants have been implicated, and the term ‘breast implant illness’ (BII) to explain this symptomatology has been coined [8].

Local complications, such as capsular contracture and allergic reactions, have been well-established with the use of silicone implants [11]. Additionally, it is hypothesized that tissue exposed to silicon can induce a host of immune reactions and inflammatory responses, which include systemic and pulmonary manifestations. Because these symptoms have often been shown to disappear after silicone implant removal, the term silicone implant incompatibility syndrome (SIIS) was used for the phenomenon [12]. Further study surrounding SIIS has identified a genetic influence underlying its development [12]. The presentation of SIIS sets it apart from the classic symptomatology of connective tissue disease and is, therefore, more aptly uniquely labeled [12,13]. To date, scientists have been unable to establish a definitive tie between the incidence of rheumatologic disease and implant use, regardless of type since doing so would require large-scale, long-duration studies [14]. We present a case of a 39-year-old female with a past medical history of breast cancer who presented to the rheumatologist for positive ANA and polyarthralgia after saline breast implants three years prior.

## Case presentation

A 39-year-old female with a past medical history of breast cancer was referred to rheumatology for positive ANA and polyarthralgia. She denied any history of alcohol or tobacco use or recent travel and was employed as a nurse. She reported being up-to-date with cervical cancer screenings. Family history was significant for Sjogren’s syndrome in her mother but negative for malignancy.

In July 2015, four years prior to her rheumatology consultation, she was diagnosed with locally advanced infiltrating ductal carcinoma of the right breast. Hormone receptor status revealed ER/PR negative and Her2/neu positive. She was started on chemotherapy with docetaxel, carboplatin, trastuzumab, and pertuzumab.

In November 2015, she underwent a bilateral mastectomy and breast reconstructive surgery and later had implantation with textured saline implants. Pathology following her mastectomy revealed ER+ positive status. She completed intrathoracic radiation therapy and a full year of trastuzumab therapy in 2016.

In July of 2016, routine imaging revealed progressive mediastinal lymphadenopathy. Biopsy demonstrated ER/PR negative and her2 neu amplification. She began therapy with trastuzumab. Despite treatment, she continued to have progressive lymphadenopathy and was later started on lapatinib and concurrent capecitabine therapy.

In June 2019, she developed fatigue, joint pain in her bilateral hands, wrists, and feet, and swelling in her fingers with concomitant morning stiffness that persisted for an hour. She was evaluated by oncology and started on a methylprednisolone dose pack, which improved her symptoms significantly. Lab work at the time revealed positive ANA 1:320 (nucleolar) and 1:160 (speckled), prompting rheumatology consultation. She had never undergone ANA testing in the past. At her rheumatology consultation in June 2019, a physical exam noted mild swelling of the bilateral metacarpophalangeal (MCP), proximal interphalangeal joint (PIP,) and distal interphalangeal (DIP) joints, difficulty making a fist, normal nailfold capillaroscopy and no evidence of skin thickening, tightening, or rash. A review of systems revealed new-onset fatigue and polyarthralgia, unintentional weight loss of five pounds, and dry eyes. Further laboratory workup revealed normal complete blood count (CBC) and comprehensive metabolic panel (CMP), mild elevation of inflammatory markers, ANA 1:640 (nucleolar) and 1:160 (speckled), highly positive U1RNP, and weakly positive chromatin and RNA polymerase III (Table 1). She had negative SSA/SSB dsDNA and Scl-70 Ab.

**Table 1:** Patient’s serum inflammatory markers

Patient’s Laboratory Workup	
ESR	31 (Normal <20)
CRP	2.6 (Normal <0.8)
ANA	1:640 (Nucleolar) 1:160 (speckled)
U1RNP76	Positive (Normal Negative)
Scl-70 Ab	Negative (Normal Negative)
RNA polymerase	Positive (Normal Negative)
Chromatin	1.3 (Cutoff less than 1)

Following an FDA-issued warning for the global recall of textured breast implants due to concerns about new cases of breast cancer involving the scar tissue surrounding the implants, the implants were removed intact in November 2019. Within days of the explantation, the patient felt that all her symptoms had resolved. Histological testing of the scar tissue on the implants tested positive for triple-positive cancer. As a result, a regimen of vinorelbine and trastuzumab was initiated, and the patient has shown good improvement in both tumor markers and follow-up imaging studies since initiation.

Postoperative physical examinations were within normal limits and the patient continued to report resolution of her previous clinical complaints. Despite the resolution of her clinical symptoms, her laboratory studies drawn four months following implant removal revealed rising titers of ANA-1:1280 (nuclear; speckled) with high inflammatory markers and persistently positive chromatin and RNA polymerase III antibodies. The patient continues to have follow-up appointments every three months for skin and joint exams and ongoing monitoring for new symptoms and follows up routinely with her managing oncologist and pulmonologist.

## Discussion

Silicone and saline implants are commonly used in breast surgery and reconstruction. However, silicone exposure, in general, has been linked to not only lung cancer and chronic renal failure but also has been shown to be related to autoimmune diseases [14]. Several factors, which can be genetic, immune, hormonal, or environmental, have been shown to increase one’s risk for autoimmune disease development [15-17]. Silicon exposure is one such environmental risk factor. Silicon is a naturally occurring element with an important role in osteogenesis [18]. Within the body, silicones are oxidized to silica, which triggers macrophage activity and cytokine and free radical production. Involvement of the lymphatic system leads to systemic inflammation [12]. Despite changes in the principal constituents of the silicone implant over the years, silicone remains an adjuvant that may ‘bleed’, thus becoming a chronic stimulus to the immune system [10]. Previous studies have hypothesized that a local immune response involving the suppression of regulatory T cells causing activation of Th1/Th17 may be involved, leading to the development of a pro-inflammatory environment and the release of cytokines. This inflammatory cascade may be the precipitating factor leading to the classic symptom pattern of BII. Additionally, more evidence of a local immunologic reaction is the association of a rare type of T-cell lymphoma, referred to as breast-implant-associated anaplastic large cell lymphoma (BIA-ALCL), which is believed to arise from the chronic inflammation surrounding the silicone breast implant [9]. With the current literature suggesting a hyperimmune state that is instigated by the SBI, it is not surprising that studies have shown an association between silicone and systemic lupus erythematosus, rheumatoid arthritis, progressive systemic sclerosis, and vasculitis [15-17].

Since the 1990s, controversy and inconsistency have been pervasive in the literature with respect to the role of breast implants in the development of autoimmune disease [11,19-21]. A small study, conducted in 1997, found that 5 of 292 breast implant recipients developed subsequent autoimmune disease, including Sjogren's syndrome (1), atypical rheumatoid syndrome (1), atypical autoimmune disease (1), and chronic fatigue syndrome (2) [22]. A 1996 study found that the relative risk of connective tissue disease in women who received breast implants compared to women who did not receive breast implants was 1.24 (95% confidence interval, 1.08 to 1.41, p=.0015) [23]. More recently, a larger study of 24,651 women who received silicone breast implants was matched with 98,604 women who did not receive implants, and found an overall 22% increase in autoimmune disorders, with Sjogren’s syndrome, systemic sclerosis, and sarcoidosis having the strongest association (OR of 1.58, 1.63 and 1.98, respectively, p < 0.001) [18]. As a result, further research is warranted to explore the association between saline breast implants and autoimmune diseases.

Studies have also shown an increased risk of positive immunological factors in women with breast illness symptoms. These serologies include antinuclear antibodies, anticardiolipin antibodies, anti-RNA polymerase III, and finally, anti-dsDNA antibodies [24]. An observational cohort study performed in Amsterdam between 2011 and 2020, investigated women with silicone breast implants and systemic symptoms that could not be explained by other causes who chose to remove their implants and compared findings to those women who chose not to have explantation. The study concluded that two-thirds of women who underwent explantation experienced moderate to significant improvement in symptoms, particularly those women who underwent explantation within 10 years of implantation. The study also observed a positive ANA in 23% of women. Previous studies examining the prevalence of positive ANA in women with SBI and systemic symptoms found variable results ranging from 5- 46%. The study went on to say that the relatively high prevalence of ANA should be interpreted cautiously as ANA prevalence is on the rise in the US, particularly in older women, thus further studies should be pursued to investigate if women with SBI truly have a higher prevalence of positive ANA [9].

In our patient’s case, her clinical symptoms improved dramatically following breast implant explantation, but her ANA titer actually increased and her chromatin and RNA polymerase III antibodies persisted. Previous studies investigating the effect of explantation and ANA found that the majority of patients continued to have positive ANA following breast implant removal. Similar to our case, some studies also found increasing titers of ANA, whereas others found persistently positive ANA with newly detectable levels of anti-centromere and anti-dsDNA. Despite the positive or newly positive serologies, the vast majority of patients had significant clinical improvement following breast implant removal [25].

## Conclusions

This clinical case of inflammatory arthritis with positive ANA antibodies following saline breast implants highlights the importance of considering the possible health implications of saline from a rheumatologic perspective. This case demonstrates that it may be reasonable that an association exists, and further research on a large scale would be valuable.
